# High perceived social support and hospital readmissions in an older multi-ethnic, limited English proficiency, safety-net population

**DOI:** 10.1186/s12913-019-4162-6

**Published:** 2019-05-24

**Authors:** Brian Chan, L. Elizabeth Goldman, Urmimala Sarkar, David Guzman, Jeff Critchfield, Somnath Saha, Margot Kushel

**Affiliations:** 1 Division of General Internal Medicine and Geriatrics, Oregon Health & Science University, 3181 SW Sam Jackson Park Road L475, Portland, OR 97239-3098 USA; 2 Division of General Internal Medicine, Zuckerberg San Francisco General Hospital, University of California, San Francisco, CA USA; 3 Division of Hospital Medicine, Zuckerberg San Francisco General Hospital, University of California, San Francisco, CA USA; 4 Central City Concern, Portland, OR USA; 5 VA Portland Health Care System, Portland, OR USA

**Keywords:** Perceived social support, Readmission, Vulnerable populations

## Abstract

**Background:**

Early readmission amongst older safety-net hospitalized adults is costly. Interventions to prevent early readmission have had mixed success. The role of perceived social support is unclear. We examined the association of perceived social support in 30-day readmission or death in older adults admitted to a safety-net hospital.

**Methods:**

This is an observational cohort study derived from the Support From Hospital to Home for Elders (SHHE) trial. Participants were community-dwelling English, Spanish and Chinese speaking older adults admitted to medicine wards at an urban safety-net hospital in San Francisco. We assessed perceived social support using the Multidimensional Scale of Perceived Social Support (MSPSS). We defined high social support as the highest quartile of MSPSS. We ascertained 30-day readmission and mortality based on a combination of participant self-report, hospital and death records. We used multiple/multivariable logistic regression to adjust for patient demographics, health status, and health behaviors. We tested for whether race/ethnicity modified the effect high social support had on 30-day readmission or death by including a race-social support interaction term.

**Results:**

Participants (*n* = 674) had mean age of 66.2 (SD 9.0), with 18.8% White, 24.8% Black, 31.9% Asian, and 19.3% Latino. The 30-day readmission or death rate was 15.0%. Those with high social support had half the odds of readmission or death than those with low social support (OR = 0.47, 95% CI 0.26–0.88). Interaction analyses revealed race modified this association; higher social support was protective against readmission or death among minorities (AOR = 0.35, 95% CI 0.16–0.76) but increased likelihood of readmission or death among Whites (AOR = 3.7, 95% CI 1.07–12.9).

**Conclusion:**

In older safety-net patients nearing discharge, high perceived social support may protect against 30-day readmission or death among minorities. Assessing patients’ social support may aid targeting of transitional care resources and intervention design. How perceived social support functions across racial/ethnic groups in health outcomes warrants further study.

**Trial registration:**

NIH trials registry number ClinicalTrials.gov: NCT01221532.

## Background

Early hospital readmissions are costly to the health care system and considered markers of poor quality; up to 20% of hospitalized patients experience readmission within 30 days [1]. The Center for Medicare and Medicaid Services (CMS) Hospital Readmissions Reduction Program has focused attention on preventing 30-day hospital readmissions [2], spurring development of transitional care interventions. These studies show mixed results [3–7], particularly in safety-net settings with higher proportions of racial/ethnic minorities, non-English speakers, and patients with lower socioeconomic status [8].

One reason for difficulties reducing readmission rates is appropriate targeting of interventions. Known clinical risk factors associated with early readmission include history of recent prior hospitalization, high burden of comorbid illness and specific diagnoses [9]. But using only these factors, predicting readmission risk remains challenging [10]. According to Andersen’s Behavioral Model of Health Services Use, predisposing factors (eg. demographics, health beliefs), enabling resources (eg. access to care, social support), in addition to need (eg. clinical status) influence utilization [11].

Social support, a social network’s provision of psychological and material resources to benefit an individual’s ability to cope with stress, may serve as an enabling resource for health services use [11, 12]. Social support may buffer against the medical, financial, and emotional stresses of hospitalization [13, 14]. While structural factors (ie. living alone, marital status) are proxies for social support, assessing one’s perceived (ie. functional) social support may better reflect health utilization risk [15–18]. But its role in readmission amongst hospitalized safety-net patients is unclear. We address this gap by examining the role of a multi-dimensional measure of perceived social support in predicting 30-day readmission rates or death in a cohort of older, multi-ethnic hospitalized adults at a safety-net hospital. We hypothesized that individuals with high social support are protected against early readmission or death.

## Methods

### Study design and sample

The study was part of a randomized controlled trial, the Support From Hospital to Home for Elders (SHHE) [7], assessing a nurse-led hospital discharge intervention in an urban, publicly-funded, acute care hospital. Details about the trial are provided elsewhere [7]. Briefly, the intervention consisted of in-person education by a trained study nurse prior to discharge, a patient-centered booklet for post-discharge care, and discharge follow-up telephone calls.

Between July 2010 and August 2012, we enrolled participants admitted to the medicine, cardiology, or neurology services of San Francisco General Hospital, an urban safety-net hospital [19]. Participants were eligible for the study if they spoke English, Spanish, or Chinese. The study was initially restricted to participants aged 60 and older, but the eligibility criteria expanded in March 2011 after 239 participants enrolled to include those aged 55 and older. We excluded participants who were [1]: transferred from an outside hospital [2]; admitted as a planned hospitalization [3]; likely to be discharged to an institutional setting [4]; unable to consent due to lack of understanding, cognitive impairment, or other reason [5]; diagnosed with metastatic cancer; and [6] unable to participate in telephone follow-up. The institutional review board of the University of California, San Francisco approved the above procedures.

### Measures

#### Data sources

We conducted language-concordant baseline interviews at bedside during hospitalization and a follow-up telephone interview 30 days after discharge. We reviewed administrative and health record data for information on comorbidities and readmissions.

#### Primary outcome

The primary outcome was 30-day readmission or death. We obtained administrative records at the index hospital and two affiliated university hospitals to ascertain readmissions data; for an additional six hospitals, we obtained administrative records for any patient who reported any hospitalization at the follow up interview. For other hospitals, we requested confirmation dates of readmission that patients reported. We identified deaths using hospital, health department, and state vital statistics records for patients who did not complete the 30-day follow up interview [7].

#### Primary predictor: perceived social support

We assessed social support using the Multidimensional Scale of Perceived Social Support (MSPSS) [20]. The MSPSS is a validated scale that asks participants 12 questions about their perceived social support (i.e., emotional support, comfort, and assistance in making decisions) from family, friends, or significant others (Table 1). Participants rated each question on a 7 point Likert like scale from “very strongly disagree” (score of 0) to “very strongly agree” (score of 7) to derive the final score up to 84. We defined social support in categories based on quartiles (5–52, 53–64, 65–74, 75+). Because the rates of 30-day readmission and death were similar amongst the lower three quartiles, we collapsed the measure into a binary predictor with scores > 74 indicating high social support, compared to those with very low, low, and medium social support.

**Table 1:** Multidimensional Scale of Perceived Social Support (MSPSS)

MSPSS Item:	Support Source Dimension
1. “There is a special person who is around when I am in need.”	Significant Other
2. “There is a special person with whom I can share my sorrows.”	Significant Other
3. “My family really tries to help me.”	Family
4. “I get the emotional help and support I need from my family.”	Family
5. “I have a special person who is a real source of comfort to me.”	Significant Other
6. “My friends really try to help me.”	Friends
7. “I can count on my friends when things go wrong.”	Friends
8. “I can talk about my problems with my family.”	Family
9. “I have friends with whom I can share my joys and sorrows.”	Friends
10. “There is a special person in my life who cares about my feelings.”	Significant Other
11. “My family is willing to help me make decisions.”	Family
12. “I can talk about my problems with my friends.”	Friends

#### Other measures

At the baseline interview, participants reported date of birth, sex, race/ethnicity (Black/African-American Non-Latino; White, Non-Latino; Latino/Hispanic, Asian, or Other), total household income (≤$20,000 per year vs > $20,000 per year) [21], last completed grade in school (≤ 12 years vs > 12 years) and limitations in activities of daily living (ADL) two weeks prior to admission (1+ ADL limitation vs none). We asked what language the participant spoke at home, and how well the participant spoke English (not at all, not well, well, and very well). We defined low English proficiency as those participants speaking English “not at all” or “not well” [22]. We measured substance use using the WHO ASSIST instrument [23] and participants self-reported any tobacco use and illicit drug use in the prior 3 months. We assessed depression at admission using the Patient Health Questionnaire-9 (PHQ-9) (we defined depression as a PHQ-9 score ≥ 10) [24], and health literacy using a self-reported 3-item scale (we defined a score ≥ 9 as inadequate health literacy) [25]. We asked participants if they lived alone, with a spouse/partner, or in another situation. We asked participants if they were hospitalized in the six months prior to enrollment. We administered the Telephone Interview for Cognitive Status (TICS) to evaluate cognitive impairment, defined as a TICS score less than 20 [26]. We calculated the Charlson co-morbidity index using administrative ICD-9 codes from the index hospitalization (scores 0, 1–2, 3–4, 5+, higher scores indicating higher co-morbidity) [27].

### Analysis

We used chi-square tests for categorical variables and t-tests for continuous variables to compare participants in the highest quartile of social support versus those in the lower three quartiles. We described the rates of high social support across White, Black, Asian, and Latino populations in the cohort.

We used multiple/multivariable logistic regression to estimate the odds ratios (and 95% CI) of 30-day readmission or death associated with the highest quartile of social support. We adjusted for variables chosen a priori based on literature or with significant association (*p* < .20) with readmission or death in bivariate analyses: demographics (age, race, sex), limited English proficiency, low health literacy, depression, cognitive impairment, any baseline ADL difficulties 2 weeks prior to admission, current smoker, hospitalized in prior 6 months, and Charlson comorbidity category. We conducted Hosmer-Lemeshow goodness of fit test to determine model fit. In a secondary analysis, we restricted the outcome to 30-day readmission and excluded patients who died to examine the effect of high social support on readmission alone.

We repeated this model using each source of social support (spouse, family, friends) as the predictor. We ran our models with and without our living situation variable to assess whether perceived social support was associated with readmission or death independent of structural support. We included treatment group assignment as a variable in the model but removed it from the final model because it was not significant. We tested for whether race/ethnicity modified the effect high social support had on 30-day readmission or death by including a race-social support interaction term. We created a new variable for White and non-White race because no Latino participants with high social support experienced 30-day readmission or death. Because the interaction was significant, we presented stratified results by race (White vs non-White).

We conducted all the analyses using Stata version 13 (StataCorp LP, College Station, TX).

## Results

### Characteristics of participants

There were 674 participants in the cohort with mean age of 66.2 years; 43.8% were women. The cohort was diverse: 24.8% were Black, 19.3% were Latino, and 31.9% were Asian. The majority of participants had annual incomes less than $20,000 (88.6%). Many reported limited English proficiency (37.2%) and had inadequate health literacy (62.7%) (Table 2). The distribution of social support using the MSPSS was skewed, with a median score of 64 (out of 84) and an interquartile range of 52–74. Compared to participants in the low social support group, those in the high social support group were more likely to be Latino (35.5% vs 14.6%) and less likely to be White (9.9% vs 21.5%). Participants with high social support were also more likely to be female, be non-smokers, and report less illicit drug use within the prior 3 months, and were less likely to have depression or live alone.

**Table 2:** Characteristics of the sample stratified by perceived level of social support (*N* = 674)

Characteristic	Total (*N* = 674) *N* (%)	High Social Support N (Col %) Total *N* = 152 (22.5%)	Low Social Support N (Col %) Total *N* = 522 (77.5%)	*p*-value
Sociodemographics				
Age, mean (±SD)	66.2 (+/− 9.0)	65.9 (+/− 8.8)	66.2 (+/− 9.0)	*P* = .71
55–59 (%)	163 (24.2)	38 (25.0)	125 (24.0)	*P* = .80
60–64	228 (33.8)	55 (36.2)	173 (33.1)	
65–74	160 (23.7)	32 (21.1)	128 (24.5)	
75+	123 (18.3)	27 (17.8)	96 (18.4)	
Female Sex	294 (43.8)	78 (51.7)	216 (41.5)	*P* = .03
Income **<** =$20,000, annually	583 (88.6)	125 (85.0)	458 (89.6)	*P* = .12
Race/Ethnicity				
Black/African-American, Non-Latino	167 (24.8)	37 (24.3)	130 (24.9)	*P* < .01
White, Non-Latino	127 (18.8)	15 (9.9)	112 (21.5)	
Latino/Hispanic	130 (19.3)	54 (35.5)	76 (14.6)	
Asian	215 (31.9)	38 (25.0)	177 (33.9)	
Education **>** 12 yrs	224 (33.2)	54 (35.5)	170 (32.6)	*P* = .50
Limited English Proficiency (LEP)	251 (37.2)	64 (42.1)	187 (35.8)	*P* = .16
Inadequate health literacy*	425 (62.7)	63 (63.6)	362 (65.5)	*P* = .83
Health-related behaviors				
Current Smoker	139 (37.9)	22 (28.6)	117 (40.3)	*P* = .06
WHO-ASSIST alcohol moderate-high risk	56 (8.0)	10 (9.8)	46 (7.71)	*P* = .47
Illicit drug use in last 3 months	42 (6.2)	3 (1.2)	39 (7.5)	*P* = .01
Health status and hospitalization factors				
Depression on admission (PHQ-9 ≥ 10)	189 (28.0)	30 (19.7)	159 (30.5)	*P* = .01
Cognitive impairment on admission (M-TICS Score < 20**)**	355 (52.7)	72 (47.4)	283 (54.2)	*P* = .14
Number of ADL Disabilities 2 weeks prior to admission				
No ADL disability	482 (71.5)	115 (75.6)	367 (76.1)	*P* = .18
1+ ADL disabilities	192 (28.5)	37 (24.3)	155 (29.7)	
Hospitalized in past 6 months	121 (18.1)	24 (15.8)	97 (18.8)	*P* = .05
Charlson Category				
0	92 (13.7)	23 (15.1)	69 (13.2)	*P* < .01
1–2	340 (50.5)	76 (50.0)	264 (50.1)	
3–4	203 (30.1)	47 (30.9)	156 (29.9)	
≥ 5	39 (5.8)	6 (4.0)	33 (6.3)	
Living situation				
Live alone or in shelter	238 (35.4)	39 (25.6)	199 (38.2)	*P* < .01
Live with spouse	388 (57.7)	84 (55.3)	304 (58.4)	*P* = .49

*Inadequate health literacy defined by self-report 3-item score ≥ 9

Abbreviations: *WHO-ASSIST* – World Health Organization – Alcohol, Smoking and Substance Involvement Screening Test; *PHQ*- Patient Health Questionnaire, *M-TICS* –Modified Telephone Interview for Cognitive Status, *ADL*- Activities of Daily Living

### Relationship between social support and 30-day readmission or death

Among the participants, 14.3% were readmitted within 30 days and 0.7% died-- a combined readmission or death rate of 15.0%. Amongst those in the highest quartile of social support, 8.6% experienced readmission or death, compared to 16.9, 14.1, and 18.1% of participants with very low, low, and medium social support. In unadjusted analyses, high social support was associated with decreased odds of 30-day readmission or death (OR = 0.47; 95% CI, 0.26–0.88). After adjustment, the association was attenuated but suggestive of a protective effect of social support (AOR = 0.63, 95% CI 0.33, 1.21) (Table 3). Hosmer-Lemeshow goodness of fit test (*p* = 0.64) showed adequate fit. Our secondary analysis examining 30-day readmission rate alone as the outcome, excluding patients who died, revealed similar results (AOR = 0.65, 95% CI 0.34, 1.24). The change in odds ratio, and loss of statistical significance, was primarily due to adjustment for race/ethnicity, which subsequent interaction analysis showed to modify the association of social support with our outcome. Adding living situation to our models did not alter our findings.

**Table 3:** Factors Associated with Readmission and death at 30 days

	Rate of Readmission or death at 30 days, %	Unadjusted OR for readmission or death at 30 days (95% CI)	Adjusted* OR for Readmission or death at 30 days OR (95% CI)
High Perceived Social Support	8.6	0.47 (0.26–0.88)	0.63 (0.33–1.21)
Age category			
55–59	14.9	Ref	Ref
60–64	14.9	0.80 (0.46–1.39)	0.69 (0.53–1.78)
65–74	12.4	0.76 (0.41–1.39)	0.83 (0.42–1.61)
75+	17.5	0.83 (0.44–1.56)	1.25 (0.59–2.66)
Female Sex	11.8	0.67 (0.43–1.03)	0.69 (0.42–1.12)
Race/Ethnicity			
Black/African-American, Non-Latino	35.3	Ref	Ref
White, Non-Latino	23.5	0.83 (0.46–1.47)	0.82 (0.43–1.57)
Latino/Hispanic	8.8	0.26 (0.12–0.57)	0.31 (0.12–0.77)
Asian	26.5	0.52 (0.30–0.90)	0.68 (0.30–1.50)
Limited English Proficiency (LEP)	12.9	1.99 (1.23–3.21)	1.38 (0.65–2.91)
Cognitive impairment on admission (M-TICS Score < 20)	16.5	1.41 (.91–2.16)	1.38 (0.84–2.26)
Depression on admission (PHQ-9 ≥ 10)	16.5	1.35 (0.86–2.12)	1.07 (0.65–1.76)
Any ADL Disability 2 weeks prior	17.4	1.54 (0.99–2.40)	1.24 (0.74–2.08)
Current Smoker	19.9	1.62 (1.00–2.62)	1.17 (0.68–2.02)
Charlson Category			
0	11.8	Ref	Ref
1–2	12.2	1.03 (0.51–2.09)	0.97 (0.47–2.02)
3–4	15.2	1.33 (0.64–2.77)	1.00 (0.46–2.20)
≥ 5	40.0	4.97 (2.04–12.12)	4.19 (1.59–11.07)
Hospitalized in the last 6 months	25.7	1.65 (1.00–2.70)	1.33 (0.76–2.32)

*Adjusted for age, sex, limited English proficiency, cognitive impairment, depression, Charlson category, ADL two weeks prior to admission, substance use, recent hospitalization, current smoker

Abbreviations: *WHO-ASSIST* – World Health Organization – Alcohol, Smoking and Substance Involvement Screening Test, *PHQ*- Patient Health Questionnaire, *M-TICS* –Modified Telephone Interview for Cognitive Status, *ADL*- Activities of Daily Living

There were differences in the sources of social support and association with 30-day readmission or death (Table 4). Participants most reported receiving high social support from a spouse (41.1%), followed by family (24.8%) and friends (21.4%). After adjustment, those who reported receiving high social support from friends had one-third the odds of experiencing 30-day readmission or death compared to those with lower levels of social support from friends (AOR, 0.32; 95% CI 0.14, 0.74). Participants with high social support from a spouse had lower 30-day readmission or death rates, but the association was not significant (AOR, 0.75; 95% CI 0.45, 1.25). Social support from family members was not associated with 30-day readmission or death (AOR, 1.10; 95% CI 0.62, 1.99).

**Table 4:** Rate of 30-day readmission or death by sources of perceived social support

Types of perceived social support	Total N (%)	Readmission or death at 30 days N (Col %) Total *N* = 102 (14.6%)	No Readmission or death at 30 days N (Col %) Total *N* = 597 (85.4%)	*p*-value
High total social support (*N* = 674)	152 (22.6)	13 (13.1)	139 (24.2)	*P* = .02
High Social support from spouse (*N* = 674)	277 (41.1)	29 (29.3)	248 (43.1)	*P* = .01
High Social support from family (*N* = 662)	164 (24.8)	21 (21.9)	143 (25.3)	*P* = .48
High Social support from friends (*N* = 639)	137 (21.4)	8 (8.5)	129 (23.7)	*P* < .01

### Interaction analysis

We found evidence that non-White vs White race/ethnicity modified the effect of high social support on odds of 30-day readmission or death (Table 5, *p*-value for interaction *p* = .002). Amongst non-Whites, those with high social support had lower odds of 30-day readmission or death compared to those with low levels of social support (AOR, 0.35; 95% CI 0.16–0.76). In contrast, Whites with high social support had higher odds of 30-day readmission or death compared to Whites with low levels of social support (AOR, 3.72; 95% CI 1.07–12.89).

**Table 5:** Interaction Analysis: Association of perceived social support with 30-day readmission or death, stratified by patient race/ethnicity

	High Perceived Social Support	Low Perceived Social Support		
	N with/without readmission or death at 30 days	N with/without readmission or death at 30 days	Unadjusted OR (95% CI) within strata)	Adjusted* OR (95% CI) within strata
White	5/10	17/95	2.79 (0.66–10.32)	3.7 (1.07–12.9)
Other (Black, Asian, Latino, Other)	8/129	69/341	0.31 (0.12–0.66)	0.35 (0.16–0.76)

*Adjusted for age, sex, limited English proficiency, cognitive impairment, depression, Charlson category, ADL two weeks prior to admission, substance use, recent hospitalization, current smoker

## Discussion

Among ethnically diverse, older adults hospitalized at a safety-net hospital, we found that those with high perceived social support had lower rates of 30-day readmission and death. After adjustment for health factors associated with readmission, the association was not significant but still suggested a protective effect of social support. We also found that while spouses are the most common sources of social support, those who perceived high social support from friends were least likely to experience early readmission and death. Finally, we found that race modified the effect of social support on 30-day readmission and death.

To date, the role of perceived social support on early readmission has been unclear. Prior studies of hospitalized heart failure patients show a positive effect of high levels of perceived social support and reduced readmission [28] but several studies of hospitalized COPD patients found no association between perceived social support and hospital readmissions at two weeks, 3 months, or 12 months [29]. A study of general hospitalized patients found no association between social support and 30-day readmission, though in that study social support was assessed with a one-item question of having someone to help at home [30]. Our findings support a role for perceived social support in preventing early readmission.

Social support may operate as an enabling resource in Andersen’s model of care utilization. In this model, utilization is not only based on need for care, but also enabling resources. Perceived social support may prevent early readmission through the ability of patients to access resources necessary for recovery from hospitalization. Our results also suggest that social support may operate via a threshold effect, because participants in the lower three quartiles of social support had similar rates of early readmission or death. Prior studies found threshold effects of social support in adjustment outcomes for breast cancer survivors [31] and perceived stress in a cohort of firefighters [32], though in contrast to our results, they found social support effects diminished at higher levels. It may be that preventing readmission requires harnessing additional sources of social support, from multiple sources. This finding is preliminary and requires additional exploration.

This study supports assessing perceived social support in addition to structural measures such as living alone. Structural social support and perceived social support are related but different concepts -- living alone is often used as a proxy for low social support but can also be a marker for good health and thereby associated with lower hospitalization risk [33]. Adjusting for living alone in our study did not substantively affect our findings. It may be that quality of support matters most in healthcare utilization, and this is best measured by evaluating how supported the patient feels. Social support is a complex concept that may not be well captured by proxy measures.

Our finding of the importance of social support from friends contradicts an early study that found social support provided by family members had stronger association with health outcomes, though that study did not assess impact on early readmission [34]. Cantor’s model of hierarchical compensation posits that older adults select from a hierarchy of supportive relationships, with spouses and family members typically selected first [35]. During times of stressful events such as hospitalization, the ability to access additional social support from an extended social network may be important post-discharge. Spousal or familial support may not be sufficient to prevent re-hospitalization when unexpected needs arise. The importance of support provided by friends may support interventions that extend support beyond the patient’s immediate family (eg community health workers).

We found that race modifies the effect of perceived social support on readmission, with high social support being protective against readmission among minorities and Latinos in particular. This may reflect how social support varies across race and culture. Multiple studies show high social support predicts improved health outcomes among Hispanics compared to non-Hispanics, and social support may contribute to the Latino health paradox phenomenon [36–38]. A study of 7374 older low-income patients discharged to home found that Black elders were more likely to report having care support, and that White elders experienced greater stress at discharge than their Black, Asian, and Latino counter parts [39]. An observational study of the National Health and Nutrition Examination Survey (NHANES) found similar interaction between non-White race and high social support and decreased odds of hypertension diagnosis [40]. On the other hand, in Whites, the role of social support and readmission is less clear. While we found an association between high social support and increased utilization, there were few patients with high social support, and subsequent wide confidence interval. In these patients, high social support may indicate higher medical acuity or social vulnerability that are not captured in the data, or that these patients’ social support appropriately directed them to seek out medical care post-discharge. Alternatively, lower levels of institutional trust in health care by non-Whites compared to Whites might also explain the differential effects of social support by race [41] -- social support may help patients disinclined to return to the hospital avoid readmission without adverse health consequences. Our findings suggest that social support matters in minorities, particularly Latinos. That few White patients reported high levels of social support compared to minorities also bears noting—White patients in safety-net populations are different medically than those in non-safety-net settings [42], and our findings may suggest they differ socially as well. The influence of perceived social support across racial/ethnic groups deserves further study.

Our findings have implications for practice. A potential reason for the mixed impact of care transitions interventions on readmission is that programs are not targeting the right patients. Medically complex safety-net patients who have high levels of social support may not need the resources these programs provide to avoid unnecessary care utilization. Prior transitional care evaluations showing efficacy such as Project BOOST (Better Outcomes for Older adults through Safe Transitions) [43] and Individualized Management for Patient-Centered Targets (IMPaCT) [44] included interventions that bolster social support of recently discharged patients in minority serving institutions; future transitions of care interventions should focus on ways of identifying patients with low social support, and then harnessing a patient’s social support network to avoid early readmission. The MSPSS can be practically administered by discharge planners to tailor transitional care planning.

### Limitations

There are limitations to this study. Our cohort was derived from those agreeing to enroll in a randomized controlled trial and may differ from the source population. The 30-day readmission rate of 14.3% was lower than anticipated and might reflect patient selection as well as the quality of care delivered during the trial period. In addition, safety-net populations differ in important ways from the general population, limiting generalizability. There may be differences in how Whites and non-Whites interpreted the social support measure. Finally, this was an observational study susceptible to confounding by unmeasured or incompletely measured variables.

## Conclusion

This study is one of the first to explore the role that perceived social support and the sources of that support have on early readmission in a cohort of low income hospitalized elders. Our results suggest that patients, particularly those of racial/ethnic minorities with high levels of perceived social support have lower odds of experiencing 30-day readmission or death. Future studies on how perceived social support functions in readmission might aid in in discharge intervention design.

## Abbreviations

ADL: Activities of Daily Living
BOOST: Better Outcomes for Older adults through Safe Transitions
CMS: Center for Medicare and Medicaid Services
ICD-9: International Classification of Disease
IMPaCT: Individualized Management for Patient-Centered Targets Involvement Screening Test
LEP: Limited English Proficiency
MSPSS: Multidimensional Scale of Perceived Social Support
NHANES: National Health and Nutrition Examination Survey
PHQ-9: Patient Health Questionnaire
SHHE: Support From Hospital to Home for Elders
TICS: Telephone Interview for Cognitive Status
WHO ASSIST: World Health Organization Alcohol, Smoking and Substance
